# Quality of life is decreased in persons with relapsing-remitting multiple sclerosis experiencing progression independent of relapse activity

**DOI:** 10.1177/13524585251318516

**Published:** 2025-02-18

**Authors:** Sarah Lindberg, Sofia Sandgren, Markus Axelsson, Igal Rosenstein, Jan Lycke, Lenka Novakova

**Affiliations:** Department of Clinical Neuroscience, Institute of Neuroscience and Physiology, Sahlgrenska Academy, University of Gothenburg, Sahlgrenska University Hospital, Gothenburg, Sweden; Department of Clinical Neuroscience, Institute of Neuroscience and Physiology, Sahlgrenska Academy, University of Gothenburg, Sahlgrenska University Hospital, Gothenburg, Sweden; Department of Neurology, Region Västra Götaland, Sahlgrenska University Hospital, Gothenburg, Sweden; Department of Clinical Neuroscience, Institute of Neuroscience and Physiology, Sahlgrenska Academy, University of Gothenburg, Sahlgrenska University Hospital, Gothenburg, Sweden; Department of Neurology, Region Västra Götaland, Sahlgrenska University Hospital, Gothenburg, Sweden; Department of Clinical Neuroscience, Institute of Neuroscience and Physiology, Sahlgrenska Academy, University of Gothenburg, Sahlgrenska University Hospital, Gothenburg, Sweden; Department of Neurology, Region Västra Götaland, Sahlgrenska University Hospital, Gothenburg, Sweden; Department of Clinical Neuroscience, Institute of Neuroscience and Physiology, Sahlgrenska Academy, University of Gothenburg, Sahlgrenska University Hospital, Gothenburg, Sweden; Department of Neurology, Region Västra Götaland, Sahlgrenska University Hospital, Gothenburg, Sweden; Department of Clinical Neuroscience, Institute of Neuroscience and Physiology, Sahlgrenska Academy, University of Gothenburg, Sahlgrenska University Hospital, Gothenburg, Sweden; Department of Neurology, Region Västra Götaland, Sahlgrenska University Hospital, Gothenburg, Sweden

**Keywords:** Multiple sclerosis, relapsing-remitting multiple sclerosis, quality of life, progression independent of relapse activity, patient-reported outcome measure, EuroQoL-5-Dimension, 29-item-MS-impact-scale

## Abstract

**Introduction:**

Reduced quality of life (QoL) is an early feature of multiple sclerosis (MS). The effect of progression independent of relapse activity (PIRA) on QoL is poorly investigated.

**Objective:**

To assess the impact of PIRA on QoL using patient-reported outcome measures (PROMs).

**Methods:**

In a prospective observational study, 125 newly diagnosed persons with relapsing-remitting MS (PwRRMS) were assessed over 5 years with: EuroQoL-5-Dimension-3-level (EQ-5D-3L), EQ-visual-analogous-scale (EQ-VAS) and 29-item-MS-Impact-Scale (MSIS-29). PwRRMS were dichotomized: PIRA (worsening of expanded disability status scale (EDSS), timed-25-foot-walk or 9-hole-peg-test, independent of relapses) versus non-PIRA. PwRRMS were compared at baseline, year 5 (y5) and delta values (baseline scores subtracted from y5 scores) and annually using linear-mixed-effects-models.

**Results:**

At y5, 19.2% had PIRA. PIRA versus non-PIRA PwRRMS were older (*p* < 0.001). At y5 PIRA PwRRMS had lower EQ-5D-3L (*p* = 0.001), higher MSIS-29-PHYS (*p* < 0.001), delta values showed lower EQ-5D-3L (*p* < 0.001) and EQ-VAS (*p* = 0.010), higher MSIS-29-PHYS (*p* = 0.004) and MSIS-29-PSYCH (*p* = 0.036). Linear-mixed-effects-models showed that, compared to PIRA, non-PIRA PwRRMS had an improvement in QoL: EQ-5D-3L: β = 0.039, *p* < 0.001; EQ-VAS: β = 2.401, *p* < 0.001; MSIS-29-PHYS: β = −0.107, *p* < 0.001; MSIS-29-PSYCH, β = −0.115, *p* < 0.001, during the 5-year study period.

**Conclusion:**

Deteriorating QoL in the early course of relapsing-remitting multiple sclerosis (RRMS) is strongly associated with PIRA. Our results suggest that QoL PROMs should be monitored and recognized as an important aspect of progression.

## Introduction

Multiple sclerosis (MS) is an inflammatory, demyelinating and neurodegenerative disorder that affects the central nervous system.^1^ Reduced quality of life (QoL) is an early feature of MS.^2^ Measuring QoL, as well as assessing other patient-reported outcome measures (PROMs), is increasingly used to make decisions in clinical practice^3^ and have been included as endpoints in clinical trials.^4^ In recent years, progression independent of relapse activity (PIRA) is recognized as the major cause of disability worsening.^5^

PIRA is common, and 25% of MS patients experience PIRA within 7 years from a first demyelinating event.^5^ It has been associated with worse long-term outcomes,^5,6^ including disability accumulation with a higher risk of reaching the expanded disability status scale (EDSS) milestone 6.^5^ For many years, the primary goal of treatment with disease-modifying therapies (DMTs) for persons with relapsing-remitting MS (PwRRMS) has been to achieve ‘no evidence of disease activity’ (NEDA-3).^7,8^ However, the available DMTs appear to have less effect on PIRA, but therapies targeting PIRA or smouldering-associated worsening^9^ are under development in MS.^10^ Although monitoring PROMs may identify disease progression in PwRRMS,^11^ the effect of early PIRA^5^ on QoL is poorly investigated. The aim of our study was to investigate the impact of PIRA on QoL in a 5-year prospective observational study in PwRRMS.

## Methods

### Study subjects

Patients were consecutively enrolled in a prospective study between 2014 and 2016 at the MS Centre, Sahlgrenska University Hospital, Gothenburg, Sweden.^12^ Eligible patients were 18 years old or older, were newly diagnosed with RRMS, and were previously untreated. Observational data were re-assessed retrospectively; patients included fulfilled the 2017 revised McDonald criteria for RRMS.^13^

All patients participated voluntarily, and written informed consent was obtained. The study conformed to the Code of Ethics of the World Medical Association (Declaration of Helsinki)^14^ and was approved by the Regional Ethics Review Board in Gothenburg, Sweden (reference number 895-13).

### Assessments

All patients were evaluated at baseline, at 6 months, and then annually throughout 5 years of follow-up. All visits included evaluation of relapse activity, disability using the EDSS,^15^ brain magnetic resonance imaging (MRI), timed 25-foot walk (T25-FW), nine-hole peg test (9HPT) and QoL assessment using PROMs. The EDSS was performed by trained neurologists in a tertiary care centre to minimize the inter-rater variability. The T25-FW and 9HPT were performed by trained study nurses.

To determine PIRA, we utilized the definition of sustained PIRA, applying a roving baseline^16^ and the following three components: EDSS,^16^ the 9HPT,^16^ and T25-FW.^16^ A PIRA event was considered if there was progression of the EDSS score (a score increase of ⩾1 if EDSS was 0.5–5.5 at baseline, or ⩾1.5 if EDSS was 0 at baseline, or ⩾0.5 if EDSS was ⩾6 at baseline), or a >20% decrease in either the 9HPT or the T25-FW test compared to the previous visit, which was confirmed at an assessment >3 months later during a period free of relapses and sustained until the end of the study at least 12 months apart from the start of PIRA.^16^ A relapse was defined as a neurological disturbance lasting longer than 24 hours, without other explanation.^17^ A period free of relapses was defined as no relapses between 90 days before and 30 days after an assessment.^16^

### QoL PROMs

The following QoL PROMs were included in the assessment: European QoL-5 Dimensions 3 levels (EQ-5D-3L),^18^ EQ visual analogue scale (VAS)^18^ and the 29-item Multiple Sclerosis Impact Scale (MSIS-29).^19^

The EQ-5D-3L index-based summary scores were generated in accordance with the National Multiple Sclerosis register guidelines using UK-based value sets^20^ and interpreted along a continuum from 1 representing best possible health, 0 representing dead, and <0 representing health worse than death.^21^ The EQ VAS ranges from 0 to 100, 0 representing worst possible health, and 100 representing the best imaginable health.^18^ The MSIS-29 consists of two subscales: physical impact (MSIS-29-PHYS, 20 items) and psychological impact (MSIS-29-PSYCH, nine items). The scores are obtained for each of the two scales by summing the responses of the individual items and then converted to a 0 to 100 range, where 100 indicates greater impact of MS.^19,22,23^

Because PROMs can identify small changes in impairment,^4^ there is a need to determine what constitutes as clinically meaningful. A minimally important difference (MID) or responder definition (RD; an individual PROM score change over a predetermined time period that should be interpreted as a treatment benefit) was used for this purpose.^4^ We defined an MID range for EQ-5D-3L of 0.050–0.084^24,25^ and for EQ VAS of 7–10.^21^ For the MSIS-29-PHYS, an RD was defined as a difference of ⩾7.5^22^ and for the MSIS-29-PSYCH, a difference of ⩾5.5.^26^

### Statistical methods

Data were analysed with descriptive statistical analyses. Continuous variables were expressed as the median and interquartile range (IQR), mean and standard deviation (SD), or mean and 95% confidence interval (CI), as appropriate. Categorical variables were expressed as frequency and percentages. Visual inspection of histograms and descriptive statistics was done to determine if outcome variables had a normal distribution.

Cross-sectional differences between PIRA versus non-PIRA patients were compared with absolute test result values at baseline and year 5 (scores from year 5 were replaced with scores from year 4 if scores from year 5 were missing; EQ-5D-3L and EQ VAS [*n* = 10], MSIS-29 [*n* = 16]). The independent sample *t*-test was used for continuous variables with normal distribution (age), the Mann–Whitney *U*-test for continuous variables that did not have a normal distribution (EQ-5D-3L, EQ VAS, MSIS-29-PHYS, MSIS-29-PSYCH, disease duration, and number of treatment changes during the study period), and the Chi-Square test for dichotomous variables (gender) and variables with three levels (treatment efficacy). The DMTs were divided into no treatment, moderate efficacy (interferon beta, glatiramer acetate, dimethyl fumarate and teriflunomide), and high efficacy (natalizumab, fingolimod, rituximab, alemtuzumab and stem cell transplantation).

For longitudinal comparison between PIRA versus non-PIRA patients, delta values, that is, change over time (baseline scores subtracted from year 5 scores) were compared using the Mann–Whitney *U*-test. Furthermore, longitudinal comparisons were done using linear mixed-effects models (LMMs) to estimate the effect of PIRA on repeated outcomes for PROMs using all available data from annual follow-up. Separate models were constructed using EQ-5D-3L, EQ VAS, MSIS-29-PHYS, and MSIS-29-PSYCH, respectively, as the dependent variable, subjects and PIRA-outcome as factors, follow-up visit and age as covariates with fixed effects of PIRA-outcome, and follow-up visit, age, interactions between PIRA-outcome and follow-up visits with each subject included as a random effect. This model allows interpretation if there is a significant effect in PROMs depending on PIRA-outcome over time for each individual PwRRMS.

To determine if differences in baseline factors and QoL PROMs remained significant when adjusted for confounding factors (age), logistic regression analysis was performed, using PIRA-outcome as the dependent variable.

All statistical analyses were performed using IBM SPSS Statistics Version 29.0.0.0 (241). All tests were two-sided, with a significance threshold of *p* < 0.05. Figures were created using GraphPad Prism 10.0.3 (GraphPad Inc., CA, USA).

## Results

### Baseline characteristics

**Figure 1.**
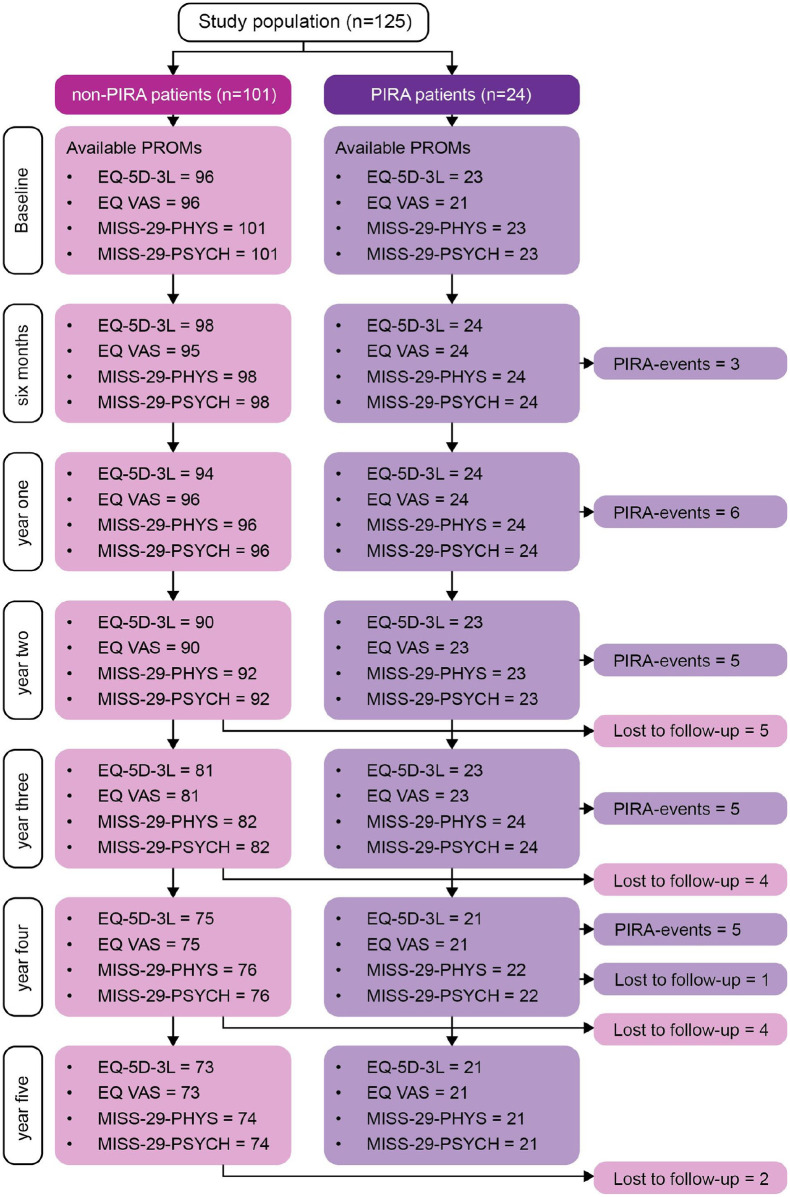
The figure shows available patient-reported outcome measures at each follow-up, when PIRA began and patients lost to follow-up. EQ-5D-3L, European quality of life-5 Dimensions 3 levels; MSIS, Multiple Sclerosis Impact Scale; PHYS, physical; PIRA, progression independent of relapse activity; PROM, patient-reported outcome measure; PSYCH, psychological; VAS, visual analogue scale.

At baseline, the study population included 125 PwRRMS (72% females), mean age 35 (SD = 10) years and median EDSS 2 (IQR 1-2.5). Twenty-four (19.2%) patients fulfilled sustained PIRA during follow-up. Patients with PIRA were older than patients with non-PIRA (mean 49 [SD = 11] vs. mean 33 [SD = 9] years, *p* < 0.001). Among PIRA patients, disability accrual was observed in EDSS in 15 (62.5%), T25-FW in five (20.8%) and 9HPT in four (16.7%) patients. There were no statistically significant differences in choice of DMT efficacy initiated at baseline or year 5 between PwRRMS with PIRA versus non-PIRA. Figure 1 shows available PROMs at each follow-up, when PIRA occurred, and patients lost to follow-up. Baseline demographics and clinical characteristics are shown in Table 1.

**Table 1.** Study population demographics and clinical characteristics.

	Total population (*n* = 125)	Non-PIRA (*n* = 101)	PIRA (*n* = 24)	*p* (non-PIRA vs. PIRA)
Age (years), mean (SD)	35 (10)	33 (9)	42 (11)	<0.001*
Gender female, *n* (%)	90 (72)	69 (68.3)	21 (87.5)	0.06
EDSS, median (IQR)	2 (1−2.5)	2 (1−2.5)	2 (1−2.37)	0.893
Disease duration (years), mean (SD)	2.21 (4.52)	2.06 (4.12)	3.04 (5.39)	0.111
Treatment efficacy initiated at baseline, *n* (%)				0.821
No treatment	8 (6.6)	6 (6.2)	2 (8.3)	
Moderate (IFNB, GLA, DMF, TFL)	67 (55.4)	55 (56.7)	12 (50)	
High (NZB, FGL, RITX, AZB, SCT)	46 (38)	36 (37.1)	10 (41.7)	
Treatment efficacy at year 5, *n* (%)				0.677
No treatment	9 (8.1)	7 (8)	2 (8)	
Moderate (IFNB, GLA, DMF, TFL)	31 (27.9)	26 (29.9)	5 (20.8)	
High (NZB, FGL, RITX, AZB, SCT)	71 (64)	54 (62.1)	17 (70.8)	
Treatment changes over the course of the study period, *n* (%)				0.805
0 changes	65 (54.2)	52 (54.2)	13 (54.2)	
1 change	44 (36.7)	34 (35.4)	10 (41.7)	
2 changes	10 (8.3)	9 (9.4)	1 (4.1)	
3 changes	1 (0.8)	1 (1)	0 (0)	

Study population characteristics, in the entire population and divided by PIRA-outcome. AZB, alemtuzumab; DMF, dimethyl fumarate; EDSS, Expanded Disability Status Scale; FGL, fingolimod; GLA, glatiramer acetate; IFNB, interferon beta; IQR, interquartile range; NZB, natalizumab; PIRA, progression independent of relapse activity; RITX, rituximab; SCT, stem cell transplantation; SD, standard deviation; TFL, teriflunomide.

Significant differences, *p* < 0.05, are marked with *.

### QoL PROMs at baseline and follow-up

At baseline, there were no significant differences between PIRA and non-PIRA patients in QoL measures.

**Figure 2.**
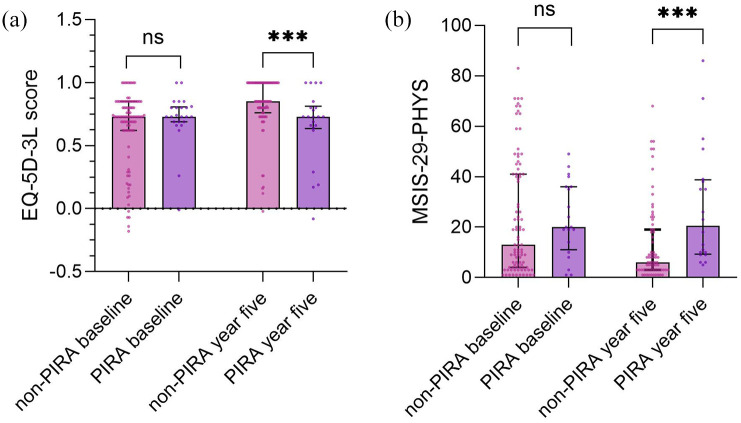
The figure shows (a), EQ-5D-3L scores and (b) MSIS-29-PHYS scores compared between non-PIRA and PIRA patients at baseline and at year 5, significant differences are marked with *. ***p < 0.001. EQ-5D-3L, European quality of life-5 Dimensions 3 levels; MSIS, Multiple Sclerosis Impact Scale; PHYS, physical; PIRA, progression independent of relapse activity; ns, not significant.

At year 5, PIRA patients had lower EQ-5L-3L (median = 0.73 [IQR = 0.64 to 0.81]) scores than non-PIRA patients (median = 0.85 [IQR = 0.76 to 1], *p* = 0.001), Figure 2(a). Furthermore, PIRA patients had higher MSIS-29-PHYS (median = 21 [IQR = 9.75 to 38.75]) scores than non-PIRA patients (median = 6 [IQR = 3 to 19], *p* < 0.001), Figure 2(b) and (Table 2).

**Table 2.** Quality of life patient-reported outcome measures as divided by progression independent of relapse activity-outcome.

	Non-PIRA (*n* = 101)	PIRA (*n* = 24)	*p*
Baseline EQ-5D-3L, median (IQR)	0.73 (0.66 to 0.85)	0.73 (0.69 to 0.81)	0.606
Year 5 EQ-5D-3L, median (IQR)	0.85 (0.76 to 1)	0.73 (0.64 to 0.81)	0.001*
Delta EQ-5D-3L, median (IQR)	0.12 (0.00 to 0.27)	0.00 (−0.15 to 0.02)	<0.001*
Baseline EQ VAS, median (IQR)	70 (50 to 85)	72 (65 to 85)	0.212
Year 5 EQ VAS, median (IQR)	77 (70 to 89)	75 (65 to 83.25)	0.285
Delta EQ VAS, median (IQR)	9 (−2.75 to 24.25)	−5 (−17 to 6)	0.010*
Baseline MSIS-29-PHYS, median (IQR)	13 (4 to 41)	20 (11 to 36)	0.524
Year 5 MSIS-29-PHYS, median (IQR)	6 (3 to 19)	21 (9.25 to 38.75)	<0.001*
Delta MSIS-29-PHYS, median (IQR)	−5 (−24 to 2)	4 (−6.25 to 16.25)	0.004*
Baseline MSIS-29-PSYCH, median (IQR)	33 (17 to 58)	33 (20.5 to 47)	0.991
Year 5 MSIS-29-PSYCH, median (IQR)	22 (8 to 36)	28 (14 to 67)	0.108
Delta MSIS-29-PSYCH, median (IQR)	−5.5 (−31 to 3)	3 (−9 to 14)	0.036*

Quality of life patient-reported outcome measures divided by PIRA-outcome at baseline, year 5 (or year 4 if results from year 5 were missing) and delta values in the study population. EQ-5D-3L, European quality of life-5 Dimensions 3 levels; IQR, interquartile range; PHYS, physical; MSIS, Multiple Sclerosis Impact Scale; PIRA, progression independent of relapse activity; PSYCH, psychological; VAS, visual analogue scale.

Significant differences, *p* < 0.05, are marked with *.

### Longitudinal change in QoL PROMs

**Figure 3.**
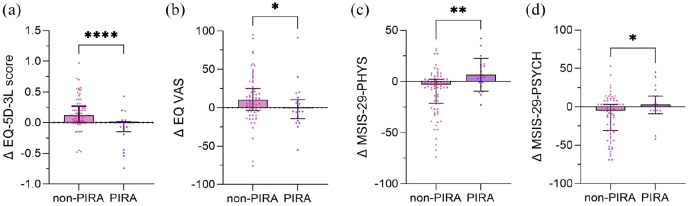
The figure shows (a) delta EQ-5D-3L scores, (b) delta EQ VAS scores, (c) delta MSIS-29-PHYS scores and (d) delta MSIS-29-PSYCH score, that is, baseline scores subtracted from year 5 scores, compared between non-PIRA and PIRA patients, significant differences are marked with *. *p < 0.05; **p < 0.01; ****p < 0.0001. EQ-5D-3L, European quality of life-5 Dimensions 3 levels; MSIS, Multiple Sclerosis Impact Scale; PHYS, physical; PIRA, progression independent of relapse activity; PSYCH, psychological; VAS, visual analogue scale.

PIRA patients had lower values for delta EQ-5D-3L (median = 0.00 [IQR = −0.15 to 0.02]) and delta EQ VAS (median = −5 [IQR = −17 to 6]) than non-PIRA patients (delta EQ-5D-3L: median = 0.12 [IQR = 0 to 0.27], *p* < 0.001, Figure 3(a); delta EQ VAS: median = 9 [IQR = −2.75 to 24.25], *p* = 0.01, Figure 3(b)), and higher values for delta MSIS-29-PHYS (median = 4 [IQR = −6.25 to 16.25]) and delta MSIS-29-PSYCH (median = 3 [IQR = −9 to 14]) than non-PIRA patients (delta MSIS-29-PHYS: median = −5 [IQR = −24 to 2], *p* = 0.004, Figure 3(c); delta MSIS-29-PSYCH: median = −5.5 [IQR = −31 to 3], *p* = 0.036, Figure 3(d)).

**Figure 4.**
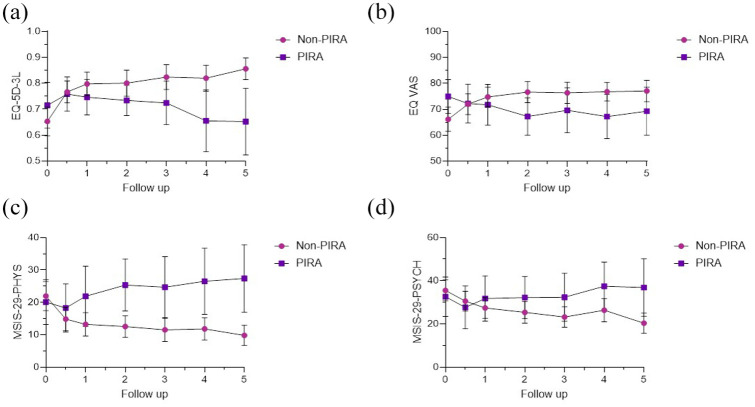
The figure shows annual mean and 95% CI for (a), EQ-5D-3L scores, and (b), EQ VAS scores, (c) MSIS-29-PHYS scores, and (d) MSIS-29-PSYCH score, for non-PIRA and PIRA patients. CI, confidence interval; EQ-5D-3L, European quality of life-5 Dimensions 3 levels; MSIS, Multiple Sclerosis Impact Scale; PHYS, physical; PIRA, progression independent of relapse activity; PSYCH, psychological; VAS, visual analogue scale.

In the LMMs, there were significant differences in QoL PROMs over time depending on PIRA-outcome. All models indicated that non-PIRA patients improved significantly in QoL measures (EQ-5D-3L: β = 0.039 [95% CI = 0.025 to 0.052], *p* < 0.001; EQ VAS: β = 2.401 [95% CI = 1.176 to 3.627], *p* < 0.001; MSIS-29-PHYS: β = −0.107 [95% CI = −0.144 to −0.070], *p* < 0.001; MSIS-29-PSYCH, β = −0.115 [95% CI = −0.168 to −0.062], *p* < 0.001, Figure 4(a)–(d)) during the 5-year study period compared to PIRA patients.

Logistic regression analysis was used to adjust for age (confounding baseline factor) between PIRA and non-PIRA patients. All variables significant in univariate analysis remained significant in logistic regression using PIRA-outcome as the dependent variable except delta MSIS-29-PSYCH (Table 3). EQ-5D-3L scores from year 5 remained significant, indicating that higher scores reduced the likelihood of having PIRA (odds ratio [OR] = 0.041, 95% CI = 0.005 to 0.341, *p* = 0.003). Delta EQ-5D-3L (OR = 0.011, 95% CI = 0.001 to 0.158, *p* < 0.001) and delta EQ VAS (OR = 0.974, 95% CI = 0.952 to 0.997, *p* = 0.027) remained significant indicating that an increase in these values reduced the likelihood of having PIRA. Year 5 (OR = 1.044, 95% CI = 1.014 to 1.075, *p* = 0.004) and delta (OR = 1.05, 95% CI = 1.013 to 1.088, *p* = 0.008) MSIS-29-PHYS remained significant, indicating that higher year 5 score and an increased delta value increased the likelihood of having PIRA. The impact from age remained significant in all models, indicating that older age made it more likely to have PIRA.

**Table 3.** Age-adjusted logistic regression models for the outcome progression independent of relapse activity.

	Univariable analysis			Multivariable analysis, separate models for each PROM, adjusted for age		
	OR	95% CI	p-value	OR	95% CI	p-value
Age	1.088	1.036 to 1.142	< 0.001*			
Year five EQ-5D-3L	0.100	0.017 to 0.592	0.011*	0.041	0.005 to 0.341	0.003*
Delta EQ-5D-3L	0.022	0.002 to 0.225	0.001*	0.011	0.001 to 0.158	< 0.001*
Delta EQ VAS	0.977	0.957 to 0.998	0.032*	0.974	0.952 to 0.997	0.027*
Year five MSIS-29-PHYS	1.038	1.012 to 1.065	0.005*	1.044	1.014 to 1.075	0.004*
Delta MSIS-29-PHYS	1.049	1.014 to 1.085	0.006*	1.05	1.013 to 1.088	0.008*
Delta MSIS-29-PSYCH	1.027	1.004 to 1.051	0.024*	1.024	1.000 to 1.050	0.051

Univariable and age adjusted individual logistic regression models using PIRA-outcome as the dependent variable for quality-of-life PROMs. CI, confidence interval; EQ-5D-3L, European quality of life-5 Dimensions 3 levels; OR, odds ratio; MSIS, Multiple Sclerosis Impact Scale; PHYS, physical; PIRA, progression independent of relapse activity; PROM, patient-reported outcome measure; PSYCH, psychological; VAS, visual analogue scale.

Significant differences, *p* < 0.05, are marked with *.

### Clinically meaningful change

Delta values for EQ-5D-3L, EQ VAS, and MSIS-29-PHYS remained significant when adjusted for age. The median delta value for non-PIRA versus PIRA patients was 0.12 versus 0 for EQ-5D-3L and 9 versus −5 for EQ VAS. The improvement in QoL that non-PIRA patients experienced was above the suggested MID thresholds.^21,24,25^ The median delta value for non-PIRA versus PIRA patients for MSIS-29-PHYS was −5 versus 4 and did not reach a difference above the RD.^22^

## Discussion

We showed that QoL measures were significantly affected in PwRRMS demonstrating early PIRA. Except for older age, PIRA patients had no other baseline clinical or demographic differences compared to non-PIRA patients, and there were no differences in baseline QoL measures. However, at the 5-year follow-up, QoL was reduced in PIRA patients.

In recent years, evidence has accumulated that even during early RRMS, PIRA is the major reason for disability accrual.^6^ Thus, the distinction between progressive MS and RRMS has been questioned and there is growing evidence that PIRA affects RRMS already from clinical onset, recently designated as smouldering-associated worsening.^9^ However, the effect of PIRA on QoL has previously not been investigated. Our results support that PIRA has significant effects on QoL in early RRMS.

The results from QoL PROMs used in our study suggest that there is a continuous impact on QoL measures in patients with PIRA. While QoL increased among non-PIRA patients, no change, or a decline in QoL was found among PIRA patients. Because year 5 and delta MSIS-29-PHYS (the physical subscale of MSIS-29) scores remained significant in a logistic regression model using PIRA-outcome as the dependent variable, but not delta MSIS-29-PSYCH (the psychological subscale of MSIS-29) scores, physical disability is more likely the main reason for the decline in QoL among patients with PIRA. Previous research shows that QoL is affected by a wide variety of symptoms with significant impact of physical disability, along with fatigue, loss of vitality and cognitive status,^2,27^ and patients with PIRA show a higher degree of disability accrual.^5^ Other studies suggest that PROMs focused on physical abilities may be successful in detecting early PIRA,^11^ as there appears to be a correlation between self-reported physical health and disease progression within 3 years.^11^ Thus, our findings suggest that the MSIS-29-PHYS subscale is a promising tool for monitoring QoL in PwRRMS with PIRA.

The median delta value for non-PIRA versus PIRA patients was 0.12 versus 0 for EQ-5D-3L and 9 versus −5 for EQ VAS. Previously, an MID range of 0.050–0.084 for US-based value sets in persons with MS^27^ and 0.074 for UK-based value sets for several other conditions^25^ were suggested for EQ-5D-3L. There is no established MID for EQ VAS for MS patients, but a range of 7 to 10 for cancer patients.^21^ Therefore, the improvement in QoL that we showed for non-PIRA patients appears clinically meaningful, as it was above the suggested MID thresholds.^21,24,25^ The median delta values for MSIS-29-PHYS also showed an increase in QoL among non-PIRA patients and a decline in PIRA patients, but did not reach a difference of ⩾7.5, which has been suggested as an RD.^22^

Although some studies show that high-efficacy DMTs have an effect on PIRA,^6,28^ other studies have failed to show such impact,^29,30^ which is in line with our results. In fact, there were no differences in DMT initiated at baseline, at year 5 or in the number of treatment switches during 5 years of follow-up between PIRA and non-PIRA patients. This could also be an indication that current surveillance of early RRMS is not sensitive enough to recognize PIRA and therefore does not lead to escalation in DMT.

There are some limitations of this study. The use of PROMs may not be appropriate for some patients and may result in inaccurately reported results and discrepancies in the estimation of symptoms and disability.^4^ All PROMs are subjective reports that can be confounded by a variety of factors.^4^ Especially EQ-5D-3L, which is a general scale, may be affected by other disorders and factors than MS. However, our results showed similar changes regardless of whether the scale was general or MS-specific. Furthermore, there were missing values from follow-up, but the statistical LMMs take these missing values into account. Furthermore, the prospective design of the study, the 5-year follow-up, and the use of LMMs minimized the bias. This study was limited to a single centre, and the effect of PIRA on QoL needs to be validated in larger studies.

In conclusion, PIRA had a negative impact on QoL PROMs in the early course of RRMS. Our results suggest that the current clinical methods used for RRMS monitoring are not sensitive to PIRA and highlight that deterioration of QoL is strongly associated with PIRA and should be monitored and recognized as an important aspect of progression. Further research is needed to evaluate the utility of QoL PROMs in PwRRMS to improve the identification of patients with PIRA.
